# Cefiderocol as a Successful Therapy for Osteomyelitis Due to XDR *Pseudomonas aeruginosa*: A Case Report and Literature Review

**DOI:** 10.3390/antibiotics14121199

**Published:** 2025-11-28

**Authors:** Alice Mulè, Anna Cambianica, Alberto Matteelli, Silvia Lorenzotti, Angelica Lenzi, Francesco Rossini, Alessio Sollima, Susanna Capone, Francesco Castelli

**Affiliations:** 1 Division of Infectious and Tropical Diseases, University of Brescia and ASST Spedali Civili Hospital, 25123 Brescia, Italy; 2 Unit of Prison Health, ASST Papa Giovanni XXIII, 24127 Bergamo, Italy

**Keywords:** cefiderocol, *Pseudomonas aeruginosa*, XDR, ESKAPE, trauma, Gram-negative, case report, bone infection, osteomyelitis, healthcare-associated infection

## Abstract

**Background**: Carbapenem-resistant *Enterobacterales* and difficult-to-treat resistance (DTR) *Pseudomonas aeruginosa* are a growing public health issue. Cefiderocol demonstrated activity against β-lactamase-producing Gram-negative bacteria (GNB). However, bone PharmacoKinetics (PK) data is lacking. Here, we report a case of post-traumatic chronic osteomyelitis caused by extensively drug-resistant (XDR) *Pseudomonas aeruginosa* which was successfully treated with cefiderocol. Moreover, we conducted a non-systematic review of the available literature. **Case Report**: We described the case of a 64-year-old man who was admitted to a traumatology ward after a work accident caused crushing of his left foot. Microbiological tests on intraoperative biopsies demonstrated XDR *P. aeruginosa* and *K. oxytoca*. Despite the administrations of different antibiotics regimens and multiple surgical revisions, the patient developed chronic osteomyelitis. To prevent amputation, cefiderocol was prescribed for six weeks, resulting in a complete clinical resolution of osteomyelitis. **Review of the Literature**: We performed a non-systematic review of the literature searching the public databases PubMed and Google Scholar. We identified nine case reports. In most patients (60%) the cause of osteomyelitis was post-surgical, and all the reported cases were healthcare associated. Osteomyelitis treatment required both antimicrobial therapy and surgery in all the cases described. Cefiderocol was often prescribed in association with other antibiotics (70%). Clinical cure was described in all the reported cases. **Conclusions**: This study highlights that cefiderocol is safe and efficacious to treat osteomyelitis caused by carbapenem-resistant GNB. However, evidence is limited to a few case reports.

## 1. Introduction

Drug-resistant Gram-negative bacteria (GNB) including carbapenem-resistant *Enterobacterales* (CRE), difficult-to-treat (DTR) *Pseudomonas aeruginosa*, and DTR *Acinetobacter baumannii* are a growing public health issue. Resistance mechanisms vary geographically and include carbapenem hydrolysis by carbapenemase enzymes, porin channel mutations, and efflux pumps overexpression [1]. Combinations of new beta-lactam/beta-lactamase inhibitors, such as ceftazidime–avibactam, ceftolozane–tazobactam, imipenem/cilastatin–relebactam, and meropenem–vaborbactam, are now available for clinical practice, but none of these are active against bacteria producing metallo-beta lactamases (MBL) [2].

Cefiderocol is a novel cephalosporin that binds to ferric iron, and it is transported through iron transport systems across the bacterial outer membrane. Once in the periplasmatic space, it binds to penicillin through Penicillin-Binding Proteins 3 (PBP-3) and inhibits peptidoglycan synthesis, causing cell death [3,4]. Cefiderocol demonstrated in vitro activity against *β*-lactamase-producing GNB (Ambler classes A, B, C, and D) [5].

In vivo studies showed that cefiderocol is safe and efficacious in the treatment of infections sustained by CRE, including serine-carbapenemases, and MBL such as verona integron-encoded metallo-beta-lactamase (VIM), and New Delhi metallo-beta-lactamase (NDM). Moreover, cefiderocol is effective against carbapenem-resistant non-fermenting bacteria such as *Pseudomonas aeruginosa*, *Acinetobacter baumannii*, *Stenotrophomonas maltophilia*, and *Burkholderia cepacia* [6,7,8]. Notably, cefiderocol may play a pivotal role in infections characterized by biofilm formation, since this process requires iron and is associated with increased siderophore production [9]. Cefiderocol has little or no activity against most Gram-positive bacteria (GPB), and, as are all cephalosporins, it is ineffective against all anaerobe bacteria.

The Federal Drug Administration (FDA) in 2019 and the European Medicines Agency (EMA) in 2020 approved cefiderocol as a therapeutic option for urinary tract infections (UTI), blood stream infections (BSI), and hospital-acquired Pneumonia (HAP) due to CRE or DTR *P. aeruginosa*. Because bone PK data after cefiderocol administration in humans are lacking, its off-label use in this context is frequent [10,11]. To date, only a single study by Mueller et al. has been published in the literature, which successfully evaluated plasma, bone, and soft tissue concentrations of cefiderocol [12]. The authors were able to demonstrate an adequate cefiderocol penetration in bones and soft tissues at 248 and 218 min after the end of a 3 h infusion of 2 g IV, when at steady state. These data need to be further confirmed to shed light on the role of cefiderocol in treating osteomyelitis caused by GNB.

We report a case of post-traumatic chronic osteomyelitis caused by extensively drug-resistant (XDR) *P. aeruginosa* which was successfully treated with cefiderocol. Furthermore, we conducted a thorough non-systematic review of the available literature on the subject, aiming to better describe and understand the efficacy and safety profile of this new molecule in the setting of osteo-articular infections.

## 2. Case Description

In February 2021, a 64-year-old man was admitted to the traumatology ward for surgical intervention on his left foot. He was involved in a work accident: a stone tile fell on his left foot while he was working as a constructor, causing exposed multi-fragmentary fractures of I, II, V metatarsal, and cuboid bones, with tarsal, tarso-metatarsal, and metatarsal-metatarsal dislocation, and laceration of the superficial and deep tissues, involving arterial and venous structures. His clinical history included vitiligo, pancreatic intraductal papillary mucinous neoplasm (IPMN) in radiologic follow-up, and previous bladder cancer treated with trans-urethral removal of the lesion some 25 years before, with radical cure. During the hospital stay reduction and fixation of the fractures with Kirshner’s wires had been performed.

In April 2021 a second surgical intervention was performed for foot reconstruction, consisting of debridement of the wound, reduction, and fixation of the fractures with Kirshner’s wires, suture of the tendons, and arterial revascularization. Microbiological tests on intraoperative biopsies demonstrated XDR *P. aeruginosa* (see antibiogram in Table 1) and pan-sensitive (PS) *K. oxytoca.* Since a thorough source control had been obtained, monotherapy with amoxicillin/clavulanic acid was prescribed for one week after surgery.

On a follow-up visit in April 2021, a Doppler ultrasound of the foot demonstrated a slow and biphasic flow on the trifurcation of the popliteal artery. The Kirshner’s wires were later removed.

In June 2021 he was admitted to the ER with a severe cutaneous ulceration of the foot and tendon exposure. After a period of Vacuum-Assisted Closure (VAC) therapy, a new surgical debridement and cutaneous grafting was performed. No microbiological test was performed on intraoperative materials, and amoxicillin/clavulanic acid was prescribed for a week after surgery.

One month later, due to skin graft rejection, surgical debridement and a new cutaneous graft were performed. Intraoperative biopsy of deep subcutaneous tissues was performed, and XDR *P. aeruginosa* and PS *K. oxytoca* were identified again; their resistance phenotype was consistent with the previous isolates. Amoxicillin/clavulanic acid was prescribed for one week after surgery aiming to eradicate *K. oxytoca*. Again, after an initial improvement, the patient developed a surgical wound dehiscence with purulent drainage from the surgical site.

In August 2021 he was admitted to the Infectious Disease ward of Spedali Civili Hospital in Brescia for worsening of the foot lesion. At the time of admission, the patient’s general conditions were stable; he was apyretic, initial laboratory analyses demonstrated mild anemia, minimal elevation of C-reactive protein (9.5 mg/L, normal value 5 mg/L), and normal white blood cells count and formula. Clinical evaluation showed an osteo-cutaneous fistula with an abundant secretion of green foul-smelling pus. A diagnosis of chronic osteomyelitis was completed.

**Figure 1:**
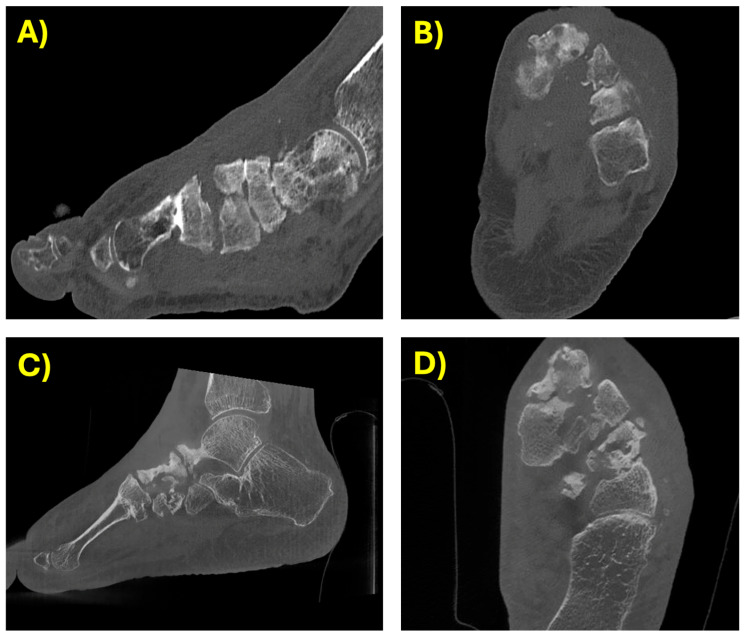
Timeline of the CT scan evolution. Panels (**A**,**B**) 19 August 2021: alteration of the dorsal bone and irregular thickening of the spongy bone of the first cuneiform, with irregular contours at the level of the joint between the first metatarsal and the first cuneiform. This finding may be compatible with osteomyelitis originating from a trophic lesion of at least 2 cm on the dorsal aspect of the midfoot. Panels (**C**,**D**) 13 January 2023: well-healed fracture of the first metatarsal, although the cortical bone on the inferior aspect, at the middle-proximal third, remains interrupted, and there is marked sclerosis of the fused fragments. The spongy bone of the midfoot remains significantly sclerotic, consistent with the sequelae of a past infection. No new erosive changes are evident.

A CT scan confirmed a severe osteomyelitis of the first metatarsal and first cuneiform bones along with anatomical subversion of the vascular and osteoarticular structures (see Figure 1); the multi-fragmentary fractures of the I metatarsal, II and III cuneiforms, and cuboid bones were unconsolidated.

XDR *P. aeruginosa* was isolated from bone biopsies (see Table 1 for antibiogram). The sample obtained from the biopsy was directly plated on Chocolate Agar, CNA, and MacConkey. Subsequently, BHI enrichment broth was used. Microbiological identification was performed using MALDI-TOF mass spectrometry (BIOMERIEUX). The antibiotic susceptibility test was conducted using the semi-automated VITEK2 system (with AST 437 card). Cefiderocol, however, was tested by microdilution using the E-TEST method.

The trauma specialists excluded any possibility of conservative interventions, proposing amputation as the only remaining surgical option.

Despite the phenotypical susceptibility, association of colistin or amikacin was not considered due to the expected poor bone penetration. Cefiderocol was initiated at the dosage of 2 g q8 h with prolonged infusion (>3 h). No adverse drug reactions were observed. During hospitalization, previously unknown arterial hypertension was diagnosed and successfully medicated with irbesartan; no correlation to cefiderocol could be demonstrated as it persisted after discontinuation of treatment. The patient was treated for 6 weeks and then discharged home.

During the follow-up the patient did not present fever or other symptoms of infection. The ulcer gradually healed after periodic dressings. His blood tests returned to normal. The CT scan excluded new erosive phenomena. The patient started a physiotherapy program that helped him to regain the ability to walk using crutches. In January 2023 a CT scan demonstrated the presence of severe surgical sequelae but did not show any sign of infection (see Figure 1). In January 2024 a final clinical evaluation confirmed a complete clinical resolution of the osteomyelitis, and the patient was able to walk without crutches.

**Figure 2:**
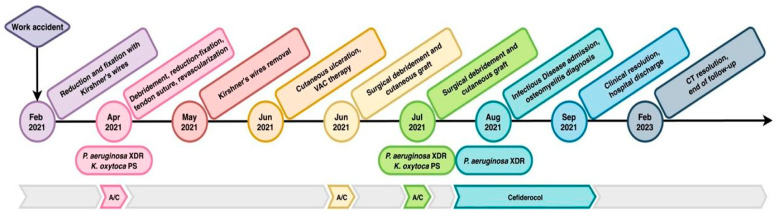
The most relevant surgical or medical events are reported on the arrow to represent their temporal sequence, microbiological isolates, and chosen antibiotic therapy are illustrated above. A/C: amoxicillin/clavulanic acid.

**Figure 3:**
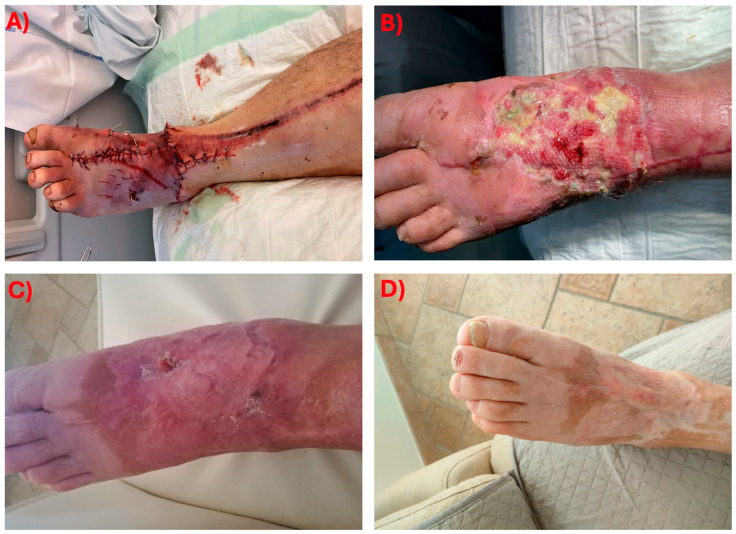
Timeline of the evolution of the foot wound. (**A**) February 2021: foot status after the first surgery of reduction and fixation of the fractures with Kirshner’s wires. (**B**) June 2021: cutaneous ulceration before the surgical debridement and skin graft. (**C**) August 2021: osteo-cutaneous fistula in chronic osteomyelitis. (**D**) Resolution of the ulceration after antibiotic treatment with cefiderocol.

Representation of the timeline of the clinical case is displayed in Figure 2, while pictures of the clinical evolution of the foot are shown in Figure 3.

## 3. Review of the Literature

### 3.1. Methods

We searched the public databases PubMed and Google Scholar with the following combination of words in the title or abstract: “cefiderocol” and “osteomyelitis” or “bone infection”. We included case series and case reports that were published up to March 2025. Also, single case description had been extrapolated by bigger case series where only one case was consistent with our search strategy. Later, we selected only the cases were *P. aeruginosa* was involved as the main or only pathogen.

Considering the major differences in clinical approach and pharmacodynamics, prosthetic joint infections and infections of surgical synthesis tools were excluded.

All sites of osteoarticular infection (including vertebral) were included in the search.

One full-text case report was not available; therefore, we reported the data contained from the abstract.

### 3.2. Results

Our search retrieved no randomized controlled trials (RCTs) or prospective studies. We identified nine case reports of patients with osteomyelitis treated with cefiderocol until March 2025.

These cases, together with the case we describe here, are summarized in Table 2.

Most patients were males (9/10; 90%) with a median age of 54.5 years (interquartile range 35).

Three patients did not present any significant comorbidity and in one case past clinical history of the patient was not available. Pre-existing pathological alteration at the site of infection, such as stage IV decubitus ulcer, was identified only in one patient.

Osteomyelitis was localized at distal leg (4/10; 40%), vertebrae (1/10; 10%), cranial (3/10; 30%), and hip/femur (2/10; 20%). Acute and chronic osteomyelitis were equally represented.

In most patients (6/10; 60%) the cause of osteomyelitis was post-surgical following a trauma. Non-traumatic origin was described in the remaining four patients.

We labeled bacterial origin as exogenous where trauma, surgery, or pre-existing pathological alterations of the site of infection were documented; other cases were considered as a result of endogenous dissemination of infection.

All the reported cases were hospital or healthcare associated.

In our review, the polymicrobial etiology exceeded the monomicrobial one (60% versus 40%, respectively). *Enterobacterales* were most frequently associated with *P. aeruginosa* (50%). Three patients presented a coinfection with GPBs (30%).

XDR *P. aeruginosa* was described as the main pathogen and the reason for cefiderocol prescription in half of the analyzed cases. Plasmidic drug-resistance mechanisms of *P. aeruginosa* described by the authors were NDM, IMP1, and VIM. Notably one pan-drug-resistant *P. aeruginosa* was described.

Bone biopsies, sometimes associated with blood cultures, allowed microbiological diagnosis in most cases (6/10; 60%). In two cases (20%) the infecting bacteria was identified in drainage fluid collected through a sterile procedure. Although the use of soft tissue swab was sometimes described, it never represented the only source for microbiological diagnosis.

Osteomyelitis treatment required both antimicrobial therapy and surgery in a combined approach in all the described cases. A conservative approach was usually preferred to demolitive surgery, but all these cases demanded multiple surgical procedures.

Cefiderocol was prescribed as a first-line therapy in a single case report, while in the other cases one or multiple antimicrobial courses preceded cefiderocol, either as empiric or targeted antimicrobial therapy. Three of them (30%) experienced some adverse drug reactions (ADRs) to the previous antibiotic regimen that required its discontinuation.

Cefiderocol was administered in association with other antibiotics in seven patients (70%). When *E. faecalis*, *S. epidermidis*, or *S. aureus* were isolated, cefiderocol was associated with antibiotics with anti-GPB activity like daptomycin or ciprofloxacin. In three cases *A. baumannii* was identified along with *P. aeruginosa,* but only in one case report cefiderocol was prescribed as a monotherapy against these two pathogens; in the remaining two cases colistin or ampicillin/sulbactam were associated with cefiderocol.

In two cases cefiderocol was associated with other anti-GNB antibiotics such as ceftazidime/avibactam and aztreonam, presumably due to the polymicrobial nature of the treated infections. Association between cefiderocol and fosfomycin was described only once.

Lastly, in two cases cefiderocol was successfully associated with phage therapy instead of traditional antibiotic therapy.

Clinical cure in absence of relapse was described in all the reported cases.

ADRs were observed in four patients (40%): two of them experienced mild and self-resolving leukopenia, one presented brown chromaturia which regressed after cefiderocol discontinuation, and one was diagnosed with relapsing *C. difficile* diarrhea.

## 4. Discussion

This report adds to the limited body of evidence regarding the efficacy and safety of cefiderocol in the management of osteo-articular infections, particularly those caused by MBL-producing pathogens.

Even if a high level of evidence on the matter is lacking, the presented case report combined with the literature review highlights the successful use of cefiderocol in the treatment of bone infections caused by XDR *Pseudomonas aeruginosa.*

In the presented case, cefiderocol was administered after the failure of previous antibiotic regimens and surgical interventions, demonstrating clinical resolution without adverse events. The review of the literature further supports the potential efficacy of cefiderocol in osteomyelitis treatment, with case reports demonstrating clinical cure in all cases. However, some safety concerns emerged, such as sporadic adverse drug reactions. Experts recommend to closely monitor the blood count during treatment and periodically rule out infective complications such as CDI [13,16,20]. As in our case, the review of the literature showed that cefiderocol is often employed as salvage therapy following failing conventional treatments [14,15,18].

Since the emergence and spreading of bacterial resistance to cefiderocol has already been reported in the literature, its use must be carefully regulated [21]. Considering that cefiderocol-resistance could lead to untreatable infections, in accordance with the most recent IDSA guidelines, we recommend keeping it as a salvage therapy, especially when other regimens are exhausted or contraindicated [22].

As in all osteomyelitis cases, source control is of paramount importance. A combined medical and surgical approach is recommended to achieve better clinical outcome, and to prevent antimicrobial resistance [23,24].

Lastly, it is important to remark that both our patient and the patients from the case reports that we examined were affected by hospital-acquired infections. In most of the cases, the infection was acquired by exogenous dissemination because of an unresolved superficial infection, rather than through the bloodstream [25].

Whether the infection sustained by these superbugs represents a complication of a previously known colonization or it is related to post-surgical factors, we can certainly consider it a direct consequence of hospitalization [26].

It is of paramount importance to raise awareness among clinicians and all healthcare workers about infection control, hands hygiene, and isolation measures, to prevent patient colonization and development of superinfections [27]. Prevention of multi-drug-resistant organism diffusion must go hand in hand with their treatment.

Our study has limitations. First, the retrospective nature of the case report and review of the literature introduces inherent biases and prevents the generalization of our findings to a broader patient population. Biases related to the tendency to publish successful cases rather than those failing treatment are also likely. Moreover, as this review is narrative rather than systematic, its reproducibility and completeness are limited. Future systematic reviews including larger cohorts will be valuable to strengthen the evidence on cefiderocol’s efficacy and safety in osteoarticular infections, and ultimately further randomized studies on larger cohorts are needed to confirm the efficacy and safety of cefiderocol in bon and joint infections caused by *P. aeruginosa*. The results of this case study should be interpreted considering previous evidence, highlighting their clinical implications and directions for future research.

Further research involving larger cohorts in randomized trials is needed to validate the efficacy and safety of cefiderocol in osteoarticular infections sustained by *P. aeruginosa*.

## 5. Conclusions

Optimal dosing, duration, and role of cefiderocol in the management of bone infections caused by DTR *P. aeruginosa* still needs to be assessed but a standard dosing of 2 g IV q8 h with prolonged infusion (>3 h) correlated to clinical cure in a few isolated clinical cases. In fact, the present case, together with the reviewed literature, supports the potential role of cefiderocol as a salvage treatment in complicated osteoarticular infections, within a multidisciplinary case management that must include surgical source control.

However, current evidence is limited to a few case reports, and further research is needed to define optimal dosing, treatment duration, and the potential for combination therapy. Given the limited body of evidence, cefiderocol should be used judiciously and within antimicrobial stewardship programs.

In conclusion, cefiderocol broadens the therapeutic landscape for bone infections caused by DTR *P. aeruginosa*, but more robust data is required to confirm these promising observations.

## Figures and Tables

**Table 1:** Antibiogram of XDR *P. aeruginosa*.

Antibiotic	MIC (mcg/mL)	Interpretation
Amikacin	4	S
Aztreonam	128	R
Cefepime	>16	R
Cefiderocol	<2	S
Cefotaxime	>32	R
Ceftazidime	>32	R
Ceftazidime/Avibactam	>8	R
Ceftolozane/Tazobactam	4	R
Ciprofloxacin	>2	R
Colistin	0.5	S
Fosfomycin	>256	R
Gentamycin	2	R
Imipenem	>8	R
Meropenem	>8	R
Piperacillin/Tazobactam	>64	R
Tobramycin	1	S

S: susceptible, R: resistant.

**Table 2:** Summary of the literature review.

(A)								
Reference	Gender, Age	Comorbidities	Onset, Site	Bacterial Origin	Sample Source	Etiology	Drug Resistance	Cefiderocol MIC
Alamarat et al. [13]	Male, 15	None	Chronic, femur	Exogenous	Bone biopsy	*P. aeruginosa*	NDM	4
						*K. pneumoniae*	ESBL	0.5
Bavaro et al. [14]	Male, 64	Hypertension, hypothyroidism	Acute, cranial	Exogenous	Surgical wound swab, bone biopsy	*P. aeruginosa*	XDR	0.5
Chavda et al. [15]	Male, 59	None	Chronic, left tibia	Exogenous	Bone biopsy, rectal swab	*P. aeruginosa*	IMP1	S
						*M. morganii*	NA	NA
						*S. epidermidis*	NA	NA
Ferry et al. [16]	Male, 74	Melanoma	Acute, vertebral	Endogenous	Bone biopsy	*P. aeruginosa*	PDR	NA
Mueller et al. [12]	Male, 48	Pyoderma gangrenosum in infliximab	Chronic, cranial	Endogenous	Blood cultures, wound swab	*P. aeruginosa*	XDR	0.5
Rose et al. [17]	Male, 50	Paraplegia, CKD, stage IV ulcer, hip dislocation	Acute, right hip and femur	Endogenous	Blood cultures, hip fluid drainage	*S. aureus*	MR	NA
						*P. mirabilis*	NA	NA
						*P. aeruginosa*	NA	NA
						*A. baumannii*	OXA-23	2
Simner et al. [18]	Male, 25	None	Acute, cranial	Exogenous	Subcutaneous drainage	*P. aeruginosa*	XDR	0.5
Smith et al. [19]	Female, 70	Diabetes mellitus type 2	Chronic, right diabetic foot	Exogenous	Soft tissues biopsies	*P. aeruginosa*	XDR	NA
						*A. baumannii*	XDR	NA
						*E. faecalis*	NA	NA
Zingg S et al. [20]	Male, 29	NA	Acute, tibia	Exogenous	Wound swab, bone biopsy	*P. aeruginosa*	VIM	NA
						*A. baumannii*	OXA-23	NA
						*E. cloacae*	KPC	NA
Current case	Male, 64	Previous bladder cancer, vitiligo, IPMN	Chronic, left foot	Exogenous	Bone and soft tissues biopsies	*P. aeruginosa*	XDR	<2
						*K. oxytoca*	PS	NA
(**B**)								
**Reference**	**Previous Therapy**	**Switch Reason**	**Cefiderocol Therapy**			**Surgery**	**Outcome. FU**	**ADR, Allergies**
			**Dose, Infusion**	**Duration**	**Concomitant Antibiotic**			
Alamarat et al. [13]	Vancomycin, cefepime, metronidazole	Inactivity	2 g q8 h, >3 h	14	Aztreonam (first 2)	Yes, conservative	Clinical cure. No recurrence at 2 mo	Yes (transient leukopenia), no
	Aztreonam, CAZ/AVI	Failure, nephrotoxicity						
	Aztreonam, colistin, tigecycline	neurotoxicity						
Bavaro et al. [14]	Meropenem, gentamycin, vancomycin	Inactivity	2 g q8 h, >3 h	2	Fosfomycin (NA)	Yes, demolitive	Clinical cure. No relapse at 30 days	No, no
	Colistin, fosfomycin	Failure						
Chavda et al. [15]	Vancomycin, ceftriaxone	Inactivity	1.5 g q8 h, >3 h 2 g q8 h, >3 h	2 2	Ciprofloxacin (4)	Yes, conservative	Clinical cure. No recurrence at 3 mo	No, no
	Vancomycin, ciprofloxacin	Failure						
	Colistin, ciprofloxacin	Nephrotoxicity						
Ferry et al. [16]	Rifampin, colistin	Nephrotoxicity, failure	2 g q8 h, >3 h 2 g q 8 h, >3 h	6	Phage therapy	Yes, conservative	Clinical cure. No recurrence at 21 mo	Yes (relapsing *C. difficile*), no
				14	Phage therapy, colistin			
Mueller et al. [12]	Ampicillin/sulbactam, doxycycline	Inactivity	2 g q8 h, >3 h	4	None	Yes, demolitive	Clinical cure. NA	No, no
Rose et al. [17]	Vancomycin	Failure	1 g q8 h *, NA 2 g q8 h, NA	6	Daptomycin (6)	Yes, demolitive	Clinical cure. *C. albicans* right hip abscess	No, no
	Daptomycin	Failure						
	Cefepime, polymyxin, eravacycline	Failure						
Simner et al. [18]	Cefepime	Inactivity	NA, NA	7.5	Phage therapy (last 4)	Yes, conservative	Microbiological, clinical cure. No recurrence at 12 mo	No, no
	IMI/REL	Failure						
	CAZ/AVI, polymyxin	Inactivity						
Smith et al. [19]	NA	NA	NA, NA	NA	Ampicillin/sulbactam	NA	NA	Chromaturia (brown), no
Zingg S et al. [20]	None	NA	NA, NA	2	CAZ/AVI, colistin (4)	Yes, conservative	Histological cure. No recurrence at 8 mo	Yes (leukopenia), no
Current case	Amoxicillin/clavulanic acid	Inactivity	2 g q8 h, >3 h	6	None	Yes, conservative	Clinical cure. No recurrence at 15 mo	No, no

NA: not available, NDM: New Delhi metallo-beta-lactamase, ESBL: extended spectrum beta-lactamase, IMP: imipenemase, S: susceptible, XDR: extensively drug-resistant, MR: methicillin-resistant, OXA: oxacillinase, KPC: *K. pneumoniae* carbapanemase, PDR: pan-drug-resistant, CKD: chronic kidney disease, IPMN: intraductal papillary mucinous neoplasm, PS: pan susceptible, CAZ/AVI: ceftazidime/avibactam, IMI/REL: imipenem/relebactam. Duration of cefiderocol or other antibiotic therapies are expressed in weeks. ADR: adverse drug reaction, * modified according to renal function.

## Data Availability

The original contributions presented in this study are included in the article. Further inquiries can be directed to the corresponding author.
